# Targeted Inhibition of Fibroblast Growth Factor Receptor 1-GLI Through AZD4547 and GANT61 Modulates Breast Cancer Progression

**DOI:** 10.3389/fcell.2021.758400

**Published:** 2021-10-13

**Authors:** Syeda Kiran Riaz, Walizeb Khan, Fen Wang, Tanwir Khaliq, Amber Malik, Eisha Tir Razia, Jahangir Sarwar Khan, Shafiul Haque, Anwar M. Hashem, Shadi S. Alkhayyat, Najiah Esam Azhar, Steve Harakeh, Mohammad Javed Ansari, Farhan Haq, Muhammad Faraz Arshad Malik

**Affiliations:** ^1^Department of Biosciences, COMSATS University, Islamabad, Pakistan; ^2^Department of Molecular Biology and Biochemistry, Shaheed Zulfiqar Ali Bhutto Medical University, Islamabad, Pakistan; ^3^College of Medicine, Texas A&M University, College Station, TX, United States; ^4^Department of Surgery, Rawalpindi Medical University, Rawalpindi, Pakistan; ^5^Research and Scientific Studies Unit, College of Nursing and Allied Health Sciences, Jazan University, Jazan, Saudi Arabia; ^6^Faculty of Medicine, Bursa Uludağ University, Bursa, Turkey; ^7^Vaccines and Immunotherapy Unit, King Fahd Medical Research Center, King Abdulaziz University, Jeddah, Saudi Arabia; ^8^Department of Medical Microbiology and Parasitology, Faculty of Medicine, King Abdulaziz University, Jeddah, Saudi Arabia; ^9^Faculty of Medicine, King Abdulaziz University, Jeddah, Saudi Arabia; ^10^General Surgery, Department of Internal Medicine, King Abdullah Medical Complex, General Directorate of Health Affairs, Jeddah, Saudi Arabia; ^11^Special Infectious Agents Unit, King Fahd Medical Research Center, King Abdulaziz University, Jeddah, Saudi Arabia; ^12^Yousef Abdullatif Jameel Chair of Prophetic Medicine Application, Faculty of Medicine, King Abdulaziz University, Jeddah, Saudi Arabia; ^13^Department of Botany, Hindu College (Moradabad), Mahatma Jyotiba Phule Rohilkhand University, Bareilly, India

**Keywords:** FGFR1, SHH pathway, GLI1, AZD4547, GANT61, breast cancer

## Abstract

The underlying mechanism of fibroblast growth factor receptor 1 (FGFR1) mediated carcinogenesis is still not fully understood. For instance, FGFR1 upregulation leads to endocrine therapy resistance in breast cancer patients. The current study aimed to identify FGFR1-linked genes to devise improved therapeutic strategies. RNA-seq and microarray expression data of 1,425 breast cancer patients from two independent cohorts were downloaded for the analysis. Gene Set Enrichment Analysis (GSEA) was performed to identify differentially expressed pathways associated with FGFR1 expression. Validation was done using 150 fresh tumor biopsy samples of breast cancer patients. The clinical relevance of mRNA and protein expression of FGFR1 and its associated genes were also evaluated in mouse embryonic fibroblasts (MEFs) and breast cancer cell line (MDA-MB-231). Furthermore, MDA-MB-231 cell line was treated with AZD4547 and GANT61 to identify the probable role of FGFR1 and its associated genes on cells motility and invasion. According to GSEA results, SHH pathway genes were significantly upregulated in FGFR1 patients in both discovery cohorts of breast cancer. Statistical analyses using both discovery cohorts and 150 fresh biopsy samples revealed strong association of FGFR1 and GLI1, a member of SHH pathway. The increase in the expression of these molecules was associated with poor prognosis, lymph node involvement, late stage, and metastasis. Combined exposures to AZD4547 (FGFR1 inhibitor) and GANT61 (GLI1 inhibitor) significantly reduced cell proliferation, cell motility, and invasion, suggesting molecular crosstalk in breast cancer progression and metastasis. A strong positive feedback mechanism between FGFR1–GLI1 axis was observed, which significantly increased cell proliferation and metastasis. Targeting FGFR1–GLI1 simultaneously will significantly improve the prognosis of breast cancer in patients.

## Introduction

The fibroblast growth factor receptor (FGFR) signaling pathway plays an important role in a variety of biological processes including angiogenesis, cell growth, differentiation, and survival (Korc and Friesel, 2009; Wesche et al., 2011). The genetic aberrations in FGFs and FGFRs linked to tumor initiation and progression are extensively reported in many cancers (Parish et al., 2015). The development of Pan-FGFR(1–4) inhibitors including ASP5878 (NCT02038673), LY2874455 (NCT01212107), infigratinib (NCT02160041), erdafitinib (NCT02365597), and AZD4547 (NCT02038673) are under different phases of clinical trials (Raja et al., 2019). However, despite of the progress, substantial effort is required to thoroughly understand the underlying mechanism of FGFR-mediated carcinogenesis. FGFs are expressed in most tissue types and play vital roles by promoting mitosis in mesenchymal and epithelial transition. In humans, 23 different FGFs have been identified, out of which 18 ligands (FGF1–10 and 16–23) are mitogenic signaling molecules (Beenken and Mohammadi, 2009). These FGFs bind and activate FGFRs (1–4), highly conserved tyrosine kinase receptors, to modulate other signaling pathways (Kwabi-Addo et al., 2004; Babina and Turner, 2017), including PLCγ/DAG/PKC, PI3K/AKT, RAS/RAF, and MAPK (Dienstmann et al., 2014; Ornitz and Itoh, 2015). FGFs also bind to heparan sulfate glycosaminoglycans (HSGAGs), which enable the activation of FGF signaling through binding FGFRs in HSGAGs-dependent manner (Beenken and Mohammadi, 2009). A recent study showed that FGF upregulation also leads to activation of SHH pathway for the ventral patterning of spinal cord (Morales et al., 2016). Moreover, upregulated FGF-FGFRs are found in many cancers including breast, prostate, non-small cell lung, liver, and colorectal (Acevedo et al., 2009; Parish et al., 2015). The genetic aberrations in FGFR1 were first documented in breast cancer. Since then, strategies are underway to regulate FGFR1-modulated cancer initiation and progression (Adnane et al., 1991). However, recent studies showed that FGFR1 upregulation causes resistance to cyclin-dependent kinase (CDK) inhibitors in different breast cancer subtypes (Formisano et al., 2019). Another study demonstrated that FGFR1 upregulation also minimize effect of other potential inhibitors targeting PI3K, ER, and mTOR pathway (Drago et al., 2019). Studies are underway to identify FGFR1-linked gene set(s) to devise effective breast cancer treatment options. For instance, a recent study showed that MAP3K1 mutation may improve the prognosis of breast cancer patients with FGFR1 overexpression (Carene et al., 2020). The aim of the current study was to identify FGFR1-linked gene sets to devise effective breast cancer treatment options. For that purpose, a comprehensive and integrated strategy was devised to establish the clinical relevance of FGFR1 modulation in breast cancer. Initially, FGFR1-expression-dependent differentially expressed pathways were identified using RNA-seq and microarray expression data of 1,425 breast cancer patients. Next, expression and clinical validation were done in 150 fresh tumor biopsy samples of breast cancer patients. Furthermore, breast cancer cell line (MDA-MB-231) was used to investigate the probable association of FGFR1 with SHH and GLI1 in breast cancer progression. The current study provides new FGFR1-linked biomarkers, which suggest novel treatment options for improving the prognosis of breast cancer patients.

## Materials and Methods

### Data Collection and Processing

The study design of the current study is described in Supplementary Material ESM 1. RNA-seq data of 1,098 breast cancer patients were downloaded from The Cancer Genome Atlas (TCGA) Database^1^. The mRNA expression levels of FGFR1 gene was estimated using z-score >2.0. The clinicopathological relevance of FGFR1 expression was assessed against multiple features including age, stage, grade, node stage, and metastasis. The details of clinical information are available in Supplementary Material ESM 2. Gene Set Enrichment Analysis (GSEA) based on FGFR1 expression was performed to identify new FGFR1-associated pathways in breast cancer (Subramanian et al., 2005). Clinicopathological association of those pathways in breast cancer was also evaluated. Additionally, another data (GSE20685) of 327 breast cancer patients were also downloaded to validate our findings in an independent cohort (Supplementary Material ESM 3). Similarly, clinical association of FGFR1 expression and FGFR1-associated pathways using GSEA was investigated. Common pathways identified between the two cohorts were selected for further analysis.

### Validation Cohort

The present study was conducted after obtaining approval from institutional ethical review committees at our university and concerned hospital. Tumor biopsies (*n* = 150) along with matched control tissues were collected for validation of discovery cohort findings after receiving informed consents of participants. Clinicopathological information of these patients was collected through subsequent follow-up from pathology reports. Information regarding clinicopathological features of validation cohort is available in Supplementary Material ESM 4.

### RNA Extraction and cDNA Synthesis

Total RNA was extracted from tumors and matched normal samples using TRIzol^®^ (Invitrogen, Carlsbad, CA, United States). cDNA was generated using RevertAid First-Strand cDNA Synthesis Kit (Thermo Fisher Scientific, Carlsbad, CA, United States) as per manufacturer’s instructions. Conventional PCR was performed with β-actin primers to confirm the cDNA synthesis. Amplified products were electrophoresed on 2% agarose gel and stained with ethidium bromide for further use.

### Primer Designing and Quantitative Real-Time PCR

Primers for FGFR1 and GLI1 were designed using Integrated DNA Technology (IDT) software and synthesized for Macrogen, Korea (Supplementary Material ESM 10). Target specificity of these products was confirmed with NCBI Primer Blast to avoid non-specific binding. Quantitative real-time PCR (qRT-PCR) quantitative PCR (qPCR) was performed using VeriQuest SYBR Green qPCR Master Mix (Thermo Fisher Scientific, CA, United States). Expression of target gene was normalized using β-actin as an internal control. The reaction condition included an initial denaturation at 95°C for 15 min, followed by 40 cycles of denaturation at 95°C for 15 s and annealing at 53°C for 1 min in each cycle. Relative mRNA expression and fold change was evaluated using the 2^–ΔΔCt^ method.

### Breast Cancer Cell Line Used in the Study

MDA-MB-231 was maintained as per American Type Culture Collection (ATCC). Expression of FGFR1, SHH, and GLI1 were assessed both at RNA and protein level. Similarly, the effect of these aforementioned molecules was evaluated on CRISPR/Cas9-mediated SHH knockout MDA-MB-231 cells (Riaz et al., 2019). Moreover, commercially available small interfering RNAs (siRNAs) targeting SHH (hs.Ri.SHH.13.1) and GLI1 (hs.Ri.GLI1.13.2) were purchased from IDT^2^, and MDA-MB-231 cells were transfected with them for assessing the differential expression of FGFR1, SHH, and GLI1.

### Exposure of GANT61 and AZD4547 Inhibitors Against SHH and GLI1

A stock of GANT61 (cat: G9048, Sigma) was dissolved in dimethyl sulfoxide (DMSO) maintaining a stock concentration of 1 mM. Briefly, 3 × 10^5^ cells were seeded in six-well plates until confluency and treated with variable concentrations of GANT61. Similarly, AZD4547 (Selleckchem^3^) was added to the medium at a concentration of 100 nM. Both RNA and protein were quantified from treated and untreated wells for functional assays performed after 48 h of treatment with both inhibitors.

### Protein Estimation Using Western Blot

Total protein content from respective cell lines were extracted and quantified using the Pierce BCA protein assay kit (23225, Thermo Fisher Scientific, United States). Extracted proteins were separated on 10% sodium dodecyl sulfate–polyacrylamide gel electrophoresis (SDS-PAGE), transferred to nitrocellulose membrane, and blocked by 5% non-fat milk at room temperature. Membranes were incubated with primary antibodies for FGFR1 (1:1,000), SHH (1:1,000), and GLI1 (1:1,000) (Supplementary Material ESM 11). After overnight incubation at 4°C, membranes were incubated with secondary antibodies at room temperature for 1 h (Supplementary Material ESM 11). Protein signals were visualized using ECL Prime Western Blotting Detection Reagent (GE Healthcare Japan) with β-actin as loading control.

### Wound Healing Assay

Briefly, 3 × 10^5^ cancer cells from respective cancer cell lines were seeded in six-well plates. The plate was left at 37°C incubation till it reaches confluent monolayer. Once attained, the medium was aspirated, and N-2-hydroxyethylpiperazine-N′-2-ethanesulfonic acid (HEPES) medium was introduced in each well. Wounding measurements were recorded using the previously mentioned protocol (Riaz et al., 2019).

### Cell Invasion Assay

This assay was based on Boyden chamber using inserts (8 μm) placed in 24-well plate. These inserts were precoated with 50 μg/ml Matrigel (BD Biosciences, Berkshire, United Kingdom) prior to cell seeding. A total of 5 × 10^4^ cancer cells were seeded in each insert placed in the respective wells. The plate was left at 37°C incubation for 24 h. After specified time duration, these inserts were fixed with methanol and stained with crystal violet. Cells were counted under light microscope at 40 × magnification as per protocol stated earlier (Malik et al., 2009).

### Statistical Analysis

IBM SPSS 21 software (IBM Inc., Armonk, NY, United States) was used for all the statistical analyses. Data were represented as mean ± SD, and Wilcoxon signed-rank test was performed to evaluate difference between tumor and control. Mann–Whitney *U* test and Kruskal–Wallis ANOVA were applied to explore any probable association of these genes with clinicopathological features. Furthermore, correlation between molecules was observed using Spearmen test. All *p*-values of 0.05 were considered statistically significant.

## Results

### Expression Analysis of Fibroblast Growth Factor Receptor 1 Gene Expression (Discovery Cohorts)

Initially, in TCGA dataset of 1,098 patients, clinical relevance of FGFR1 expression with breast cancer patients was assessed. FGFR1 was significantly overexpressed in late tumor stage (*p* = 0.05) and node-positive patients (*p* = 0.04) (Table 1).

**TABLE 1:** Clinicopathological analysis of *FGFR1* and *GLI1* in discovery cohort 1.

Variables	Total	Mean ± SD			
		
		*FGFR1*	*p*-value	*GLI-1*	*p*-value
Samples	1,091	6.69 ± 1.21	0.0001^c^	−0.11 ± 1.57	0.0001^c^
**Stage-wise distribution**					
Stage I/II	910	6.68 ± 1.21	–	−0.13 ± 1.56	0.0001^b^
Stage III/IV	177	6.73 ± 1.22		−0.001 ± 1.63	
**Nodal Involvement**					
N0 (none)	874	6.66 ± 1.24	0.002^b^	−0.12 ± 1.56	0.044^b^
Nodal metastasis	196	6.81 ± 1.04		−0.04 ± 1.62	
**Metastasis involvement**					
M0	907	6.66 ± 1.22	–	−0.17 ± 1.55	0.022
M1	22	6.77 ± 1.27		−0.96 ± 1.99	
**Age group**					
Above 50	760	6.69 ± 1.10	–	0.23 ± 1.54	0.0001^a^
Below 50	329	6.73 ± 1.25		−0.26 ± 1.57	

*^*a*^Mann–Whitney test.*

*^*b*^Kruskal–Wallis test.*

*^*c*^Wilcoxon signed-rank test.*

### Fibroblast Growth Factor Receptor 1-Expression-Based Gene Set Enrichment Analysis

Next, GSEA was performed to identify common differentially expressed pathways in both breast cancer cohorts. A total of 20 pathways were found to be upregulated along with FGFR1 overexpression in TCGA dataset (Supplementary Material ESM 5). Similarly, a total of 14 pathways were found to be upregulated along with FGFR1 overexpression in the GEO20685 cohort (Supplementary Material ESM 6). Out of all pathways, three pathways including ALK, SHH, and PRION were common in both datasets (Supplementary Material ESM 7). Since SHH pathway dysregulation is reported as an early event in several breast cancer studies, we selected SHH pathway to evaluate the association of FGFR1 and SHH pathway in modulating breast carcinogenesis.

### Clinicopathological Relevance of SHH Pathway Genes

**FIGURE 1:**
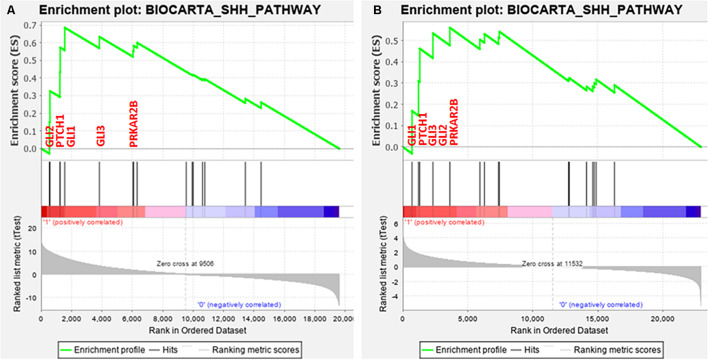
Enrichment plot: BIOCARTA_SHH_PATHWAY of both datasets. **(A)** TCGA and **(B)** GSE20685 that shows profile of the running ES score and positions of gene set members on the rank ordered list.

Interestingly, 5 out of 16 SHH pathway genes showed core enrichment in FGFR1-expressed breast cancer patients using leading-edge subset method (Figure 1 and Supplementary Material ESM 8). Of note, all GLI family genes including GLI1, GLI2, and GLI3 were significantly associated with FGFR1 in both datasets. Therefore, to further establish the link of FGFR1 overexpression with GLI genes, clinicopathological association of genes including FGFR1, GLI1, GLI2, and GLI3 were evaluated (Supplementary Material ESM 9). According to the results, GLI1 gene was the most frequently associated gene with the poor prognostic features of breast cancer patients including late stage (*p* = 0.0001), node positive (*p* = 0.044), and metastasis (*p* = 0.022) (Table 1 and Supplementary Material ESM 9). Similarly, in the second dataset of 327 breast cancer patients, GLI1 overexpression was the only gene in the SHH pathway that showed significant associations with late stage (*p* = 0.047). Therefore, based on all the expression and statistical analyses in discovery cohorts, we further established the potential prognostic association of GLI1 and FGFR1 genes using normal and tumor pairs of 150 breast cancer patients.

### *In vitro* Validation of Fibroblast Growth Factor Receptor 1 and GLI1 Association in 150 Breast Cancer Patients

The demographics and clinical characteristics of the cohort exhibited that the mean age of breast-cancer-affected patients included in the study was 45 years, ranging from 23 to 75 years. According to Wilcoxon test, both FGFR1 (*p* = 0.0001) and GLI1 (*p* = 0.0001) were significantly overexpressed in tumor samples compared to their respective controls. Interestingly, FGFR1 and GLI1 expression showed strong positive correlation (Spearman’s correlation = 0.513, *p* = 0.0001). Of note, consistent with discovery cohort findings, overexpression of GL1 and FGFR1 genes was significantly associated with high grade (*p* = 0.05), late stage (*p* = 0.011, *p* = 0.004), and metastasis (*p* = 0.006, *p* = 0.04), respectively (Table 2). These findings suggest strong biological and prognostic relevance of GLI1 and FGFR1 expression in modulating subtypes of breast cancer progression.

**TABLE 2:** Clinicopathological analysis of *FGFR1* and *GLI1* expression in *in vitro* cohort.

Variables	Total	Mean ± SD			
		
		*FGFR1*	*p*-value	*GLI-1*	*p*-value
Tumor	150	18.6 ± 26	0.0001^a^	20.5 ± 28.96	0.0001^a^
Control	150	1 ± 1.41	–	1 ± 1.95	–
**Grade-wise distribution**					
Grade I	14	22.71 ± 34.69	–	9.27 ± 11.55	0.05^b^
Grade II	90	15.65 ± 19.3		20.11 ± 29.76	
Grade III	46	23.12 ± 33.41		24.74 ± 30.48	
**Stage-wise distribution**					
Stage I/II	111	16.12 ± 21.31	0.004^b^	17.29 ± 24.22	0.011^b^
Stage III/IV	39	25.65 ± 35.69		29.71 ± 38.38	
**Nodal involvement**					
N0 (none)	48	16.86 ± 23.49	–	20.06 ± 32.80	–
Nodal metastasis	102	19.42 ± 27.23		20.73 ± 27.14	
**Metastasis involvement**					
M0	145	17.97 ± 26	0.040^b^	19.67 ± 28.88	0.006^b^
M1	5	36.66 ± 22.39		45.17 ± 20.9	

*^*a*^Wilcoxon Signed-ranks test.*

*^*b*^Mann–Whitney test.*

### Effect of GANT61 and AZD4547 Treatment on Fibroblast Growth Factor Receptor 1-GLI1 Expression in MDA-MB231

**FIGURE 2:**
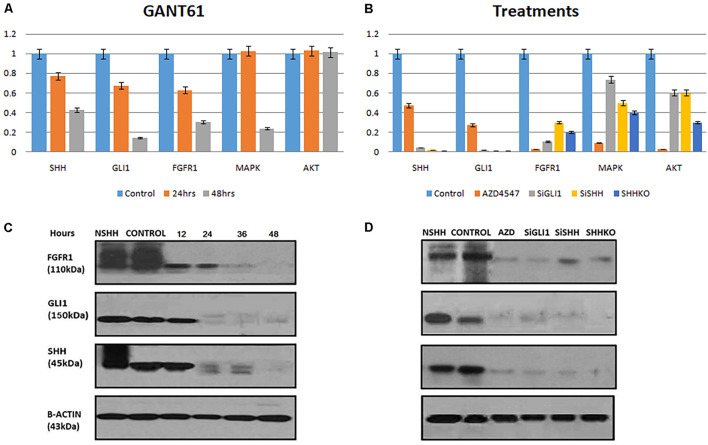
Synergistic effect of FGFR1 and Hedgehog signaling in breast cancer cells. **(A)** MDA-MB-231 cells were treated with 10 μM GANT61, and samples were collected after 24 and 48 h of treatment. Significant decrease in mRNA expression of FGFR1 was observed after treatment with GANT61 at 24 and 48 h both, while expression of MAPK was reduced after 48 h of treatment. No effect of GANT61 treatment was observed on transcription of AKT in MDA-MB-231 cells. **(B)** Effect of FGFR1 inhibitor [AZD4547 (0.2 nM)], GLI1 knockdown (SiGLI1), SHH knockdown (SiSHH), and SHH knockout (SHHKO) on SHH, GLI1, FGFR1, MAPK, and AKT at transcriptional level. Cells were collected after 48 h of treatment or gene silencing. Western blot showing decrease in expression of FGFR1, GLI1, and SHH upon treatment with **(C)** GANT61 (10 μM) administered until 48 h with a 12-h interval and **(D)** AZD4547 (0.2 nM), SiGLI1, SiSHH, and CRISPR/Cas9-mediated SHH knockout in MDA-MB-231 breast cancer cells. Cells were collected 48 h after treatment AZD4547, SiGLI1, and SiSHH. SHH ligand (NSHH) was also added to MDA-MB-231 cells to observe effect of pathway induction. B-Actin was used as internal control.

Next, the effect of GANT61 (GLI1 inhibitor) and AZD4547 (FGFR1 inhibitor) on both GLI1 and FGFR1 was studied in MDA-MB-231 cell line. To perform the analysis, multiple genes including SHH, AKT, and MAPK were also selected to compare the effect. According to the results, a significant decrease in the expression of GLI1, SHH, and FGFR1 was observed after treatment with GANT61 at 24 and 48 h. MAPK expression was reduced after 48 h of treatment. However, no change in the expression of AKT gene was observed (Figure 2A). Interestingly, a significant downregulation of FGFR1 expression was observed compared to other genes when cells were treated with AZD4547, GLI1 knockdown (SiGLI1), SHH knockdown (SiSHH), and SHH knockout (SHHKO), suggesting a crosstalk between SHH pathway and FGFR1 activation (Figure 2B).

### Synergistic Effect of Fibroblast Growth Factor Receptor 1 and Hedgehog Signaling

**FIGURE 3:**
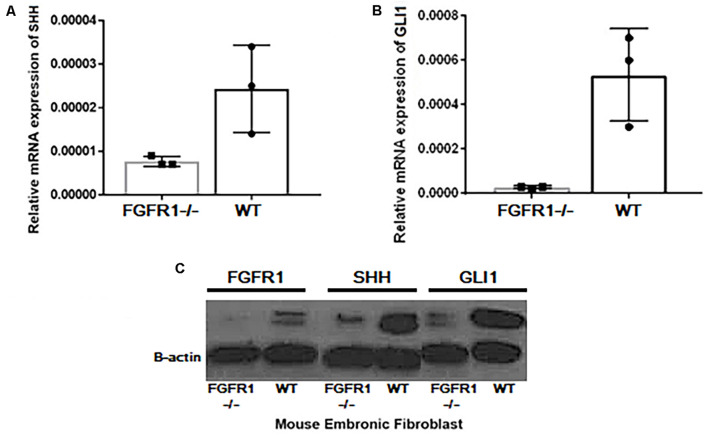
Expressional variation of SHH and GLI1 at transcript level in MEF^WT^ and MEF^FGFR1 KO (–/–)^. Significant downregulation of **(A)** SHH and **(B)** GLI1 at mRNA and **(C)** protein level was observed in the FGFR1 knockout (−/−) and wild-type (WT) mouse embryonic fibroblast cells (MEFs) showing crosstalks between both pathways.

Next, using Western blot analysis, the impact of GANT61, AZD4547, SiGLI1, and SiSHH was assessed in MDA-MB-231 cells on SHH, GLI1, and FGFR1 expression. First, GANT61 treatment was administered until 48 h with 12-h intervals. A significant decrease in protein expression was observed after 12 h for all three genes including SHH, GLI1, and FGFR1 (Figure 2C). In addition, AZD4547, SiGLI1, SiSHH, and CRISPR/Cas9-mediated SHH knockout in MDA-MB-231 breast cancer cells was also assessed (Figure 2D). Consistently, a similar pattern of decrease in protein expression was observed for SHH, GLI1, and FGFR1, suggesting the synergistic role of FGFR1 and SHH pathway. Furthermore, we also evaluated the effect of SHH and GLI1 expression in mouse embryonic fibroblasts (MEFs) with MEFFGFR WT and MEFFGFR1 KO cells. Significant downregulation of SHH and GLI1 was observed in the FGFR1 knockout mouse MEFs, further indicating crosstalks between FGFR–SHH pathways (Figure 3).

### Combined Treatment of GANT61 and AZD4547 Reduces Cell Motility and Invasion

**FIGURE 4:**
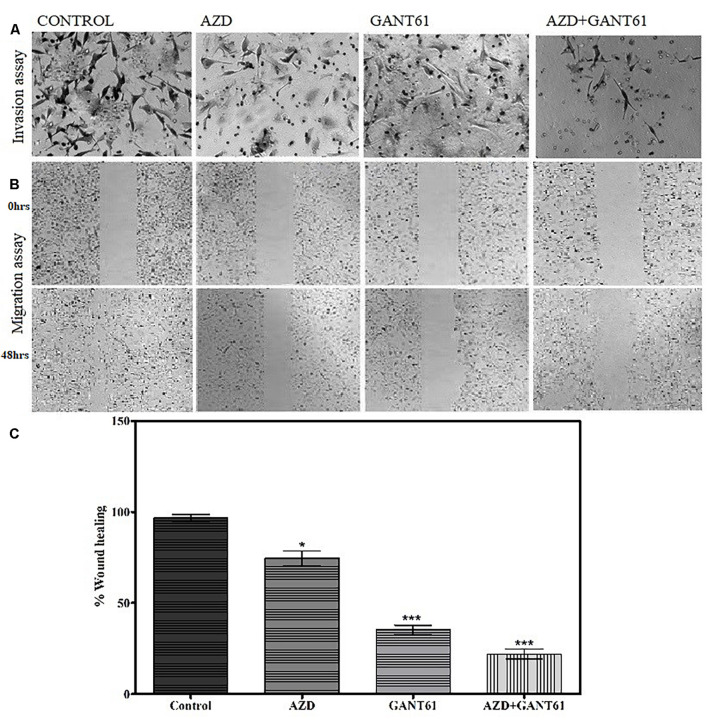
Decrease in migratory and invasive abilities of breast cancer cells was observed upon inhibition of FGFR1-GLI1 using *in vitro* models. **(A)** Invasion and **(B)** migration assay was performed in control cells, AZD4547 (0.2 nM)-treated cells, GANT61 (10 μM)-treated cells, and AZD + GANT61 combined treatment in MDA-MB-231 cells (scale bar, 50 μm). **(C)** Histogram showing overall difference in migration of cells after 48 h of AZD4547, GANT61, or combined treatment. Invasion and migration of MDA-MB-231 decreased significantly upon AZD + GANT61 treatment (ANOVA with Dunnett’s *post hoc* test, *p < 0.05, ***p < 0.0001). All results are representative of three independent experiments.

Furthermore, the effect of FGFR1–SHH pathway signaling on the motility of breast cancer cells was evaluated using wound-healing assay. MDA-MB-231 cells were treated with AZD4547 only, GANT61 only, and AZD + GANT61 combined (Figure 4). Breast cancer cell migration was evaluated after 48 h of treatment. Of note, invasion and migration of MDA-MB-231 decreased significantly upon AZD + GANT61 treatment compared to AZD4547 or GANT61 treatment alone. Therefore, the data suggest that targeting FGFR1–GLI1 simultaneously significantly reduce cell invasion and metastasis.

## Discussion

*Fibroblast Growth Factor Receptor 1* DNA amplification is the most frequently reported FGF-pathway alteration in breast cancer (Sobhani et al., 2020). *FGFR1* is a known poor prognostic marker also associated with poor survival in breast cancer patients (Wu et al., 2018). Herein, we reported a different mechanism by which coexpression of *FGFR1* and *GLI1* genes leads to breast cancer invasion and metastasis. We also demonstrated that simultaneous targeting of FGFR1 and GLI1 using GANT61 and AZD4547 inhibitors significantly decreases breast cancer invasion and metastasis, suggesting new approaches for clinical studies. The dysregulation of Hedgehog signaling, including GLI1 upregulation, is frequently reported in young breast cancer patients with shorter overall survival (Riaz et al., 2018). Of note, FGFR–Hedgehog pathway crosstalk showed variable prognostic roles in different cancers. For instance, in non-small cell lung cancer, FGFR1–GLI1 axis promotes lung carcinogenesis (Ji et al., 2016). On the contrary, preclinical study in medulloblastoma showed antagonistic role between FGFR1 and GLI1 expression, suggesting tissue-specific role of these pathways (Neve et al., 2019). However, crosstalk between FGFR and Hedgehog signaling is not elucidated in breast cancer. Here, using large-scale transcriptomic data of 1,425 breast cancer patients, we demonstrated that FGFR/SHH pathway crosstalk leads to poor prognosis of breast cancer patients. Particularly, FGFR1 and GLI1 showed strong correlation with late stage, node, and metastasis in luminal subtypes of breast cancer patients.

These observations were further validated using qPCR method in 150 paired tumor-normal cases. The results showed strong correlation (*p* < 0.5) between FGFR1 and GLI1 and significant association with late stage, high grade, and metastasis, further emphasizing on the FGFR1–GLI1 nexus in breast carcinogenesis. Next, the prognostic impact of FGFR1–GLI1 axis inhibition was evaluated. Cyclin-dependent kinase 6 (CDK6) inhibitors are the most widely used treatment options in breast cancer patients (Parish et al., 2015). However, it has been observed that FGFR1 upregulation causes resistance to CDK inhibitors in subtypes of breast cancer patients, suggesting alternate treatment strategies (Formisano et al., 2019). Recently, it has been demonstrated that treatment with multiple inhibitors may improve the prognosis and increase the survival of breast cancer patients (Slamon et al., 2020). For that purpose, the efficacy of AZD4547, a selective inhibitor of FGFR1, and GANT61, a GLI1 inhibitor, was evaluated in FGFR1–GLI1-overexpressed MDA-MB-231 cell line. Interestingly, the results showed coexpression of FGFR1 and GLI1, suggesting molecular crosstalk between both genes (Figure 1). Moreover, inhibiting either FGFR1 or GLI using AZD4547 or GANT61, respectively, also decreases expression of both genes (Figure 2). Furthermore, combined inhibition of FGFR1–GLI1 with AZD + GANT61 inhibitors drastically decreased the migratory and invasive abilities of breast cancer cells, suggesting a novel mechanism to treat breast cancer patients. In conclusion, a comprehensive and integrated strategy was devised to find new therapeutic options for the treatment of breast cancer. Clinical analyses of whole transcriptomic data and multiple *in vitro* functional assays strongly suggested that targeting FGFR1–GLI1 crosstalk can significantly improve the prognosis of breast cancer. Interestingly, FGFR1 and GLI1 also activate many downstream oncogenic genes, which lead to cancer cell migration, proliferation, and survival (Luca et al., 2017; Riaz et al., 2018). Hence, our study provided a novel hotspot target site as plausible therapeutic option for breast cancer treatment.

## Data Availability Statement

The original contributions presented in the study are included in the article/Supplementary Material, further inquiries can be directed to the corresponding authors.

## Ethics Statement

The studies involving human participants were reviewed and approved by the ERB Shaheed Zulfiqar Ali Bhutto. The patients/participants provided their written informed consent to participate in this study. The animal study was reviewed and approved by the Ethics Review Board of the Texas A&M University.

## Author Contributions

SKR, WK, FW, AM, TK, ER, JK, ShH, AH, SA, NA, StH, FH, and MFM: conceptualization, visualization, and writing—original draft. SKR, WK, FW, TK, AM, ER, and JK: data curation. SKR, WK, FW, TK, AM, ER, JK, FH, and MFM: formal analysis, methodology, and validation. FW, FH, and MFM: funding acquisition. SKR, ER, TK, JK, ShH, AH, SA, NA, StH, FH, and MFM: investigation. ShH, AH, SA, NA, StH, FH, and MFM: project administration. ShH, AH, StH, FH, and MFM: resources and supervision. FH and MFM: software. ShH, AH, StH, FH, and MFM: writing—review and editing. All authors contributed to the article and approved the submitted version.

## Conflict of Interest

The authors declare that the research was conducted in the absence of any commercial or financial relationships that could be construed as a potential conflict of interest.

## Publisher’s Note

All claims expressed in this article are solely those of the authors and do not necessarily represent those of their affiliated organizations, or those of the publisher, the editors and the reviewers. Any product that may be evaluated in this article, or claim that may be made by its manufacturer, is not guaranteed or endorsed by the publisher.

## Footnotes

1
https://portal.gdc.cancer.gov/projects/TCGA-BRCA

2
https://sg.idtdna.com/pages

3
https://www.selleckchem.com/products/azd4547.html
