# Supplementary material for: Targeted Inhibition of Fibroblast Growth Factor Receptor 1-GLI Through AZD4547 and GANT61 Modulates Breast Cancer Progression
Source: Front Cell Dev Biol. 2021 Oct 13;9:758400. doi: 10.3389/fcell.2021.758400 (PMC8548881; doi:10.3389/fcell.2021.758400)
Supplement: Supplementary file 1 [file Data_Sheet_1.docx]

Supplementary Material

**ESM 1 Study Design**


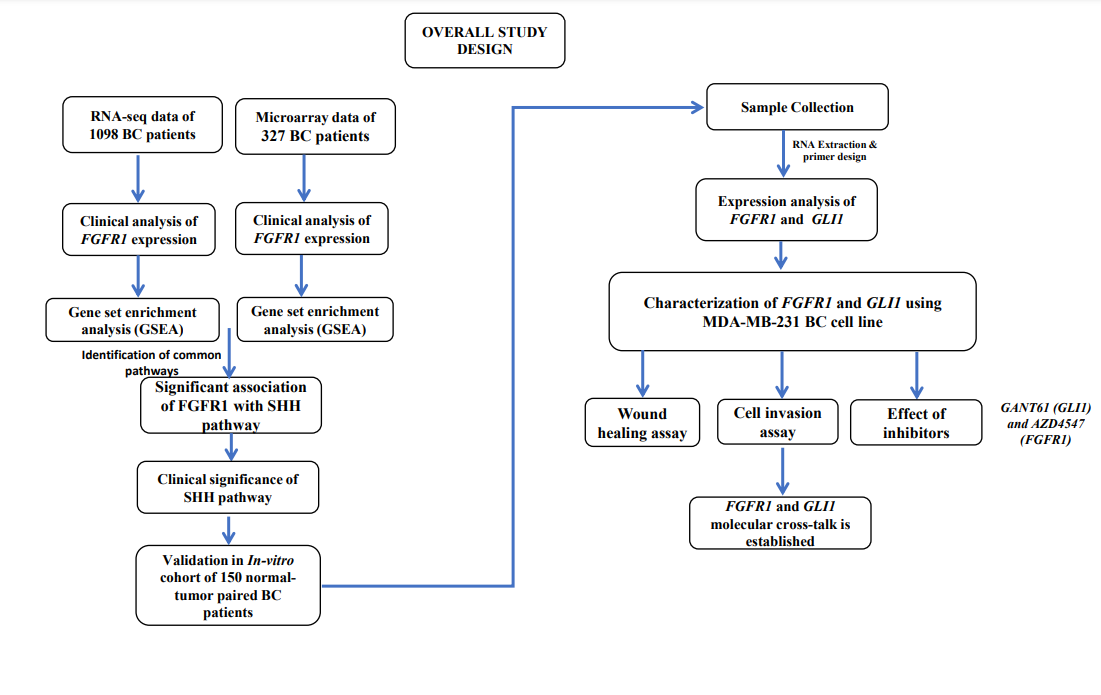


**ESM. 2:** Clinico-pathological features of TCGA dataset.

| **Variable** | **Number** | **Total** |
| --- | --- | --- |
| **Samples** | 1091 | 1091 |
| **Stage-wise distribution** | | 1087 |
| **Stage I/II** | 910 |  |
| **Stage III/IV** | 177 |  |
| **Nodal Involvement** | | 1070 |
| **N0 (none)** | 874 |  |
| **Nodal metastasis** | 196 |  |
| **Metastasis Involvement** | | 929 |
| **M0** | 907 |  |
| **M1** | 22 |  |
| **Age Group** | | 1089 |
| **Above 50** | 760 |  |
| **Below 50** | 329 |  |
| **Molecular subtypes** | |  |
| **ER +** | 803 | 1040 |
| **ER -** | 237 |  |
| **PR +** | 694 | 1037 |
| **PR -** | 343 |  |
| **HER2 +** | 78 | 408 |
| **HER2 -** | 330 |  |

**ESM. 3:** Clinico-pathological features of GEO20685 dataset.

| **Variable** | **Total** |
| --- | --- |
| **Samples** | 327 |
| **Stage-wise distribution** | |
| **Stage I/II** | 289 |
| **Stage III/IV** | 38 |
| **Nodal Involvement** | |
| **N0 (none)** | 137 |
| **Nodal metastasis** | 190 |
| **Metastasis Involvement** | |
| **M0** | 319 |
| **M1** | 8 |
| **Age Group** | |
| **Above 50** | 208 |
| **Below 50** | 118 |

**ESM. 4:** Clinico-pathological features of in-vitro cohort dataset.

| **Variable** | **Total** |
| --- | --- |
|  |  |
| **Tumor** | 150 |
| **Control** | 150 |
| **Grade-wise distribution** | |
| **Grade I** | 14 |
| **Grade II** | 90 |
| **Grade III** | 46 |
| **Stage-wise distribution** | |
| **Stage I/II** | 111 |
| **Stage III/IV** | 39 |
| **Nodal Involvement** | |
| **N0 (none)** | 48 |
| **Nodal metastasis** | 102 |
| **Metastasis Involvement** | |
| **M0** | 145 |
| **M1** | 5 |
| **Age Group** | |
| **Above 45** | 74 |
| **Below 45** | 76 |
| **Molecular subtypes** | |
| **ER +** | 73 |
| **ER -** | 77 |
| **PR +** | 94 |
| **PR -** | 56 |
| **HER2 +** | 62 |
| **HER2 -** | 88 |
|  | |

**ESM. 5:** 20 pathways associated with FGFR1 overexpression in TCGA

| **List of Pathways which show significance with Up-Regulation of FGFR1 in TCGA dataset** | |
| --- | --- |
| BIOCARTA_VIP_PATHWAY | BIOCARTA_BARRESTIN_PATHWAY |
| BIOCARTA_PRION_PATHWAY | BIOCARTA_SPRY_PATHWAY |
| BIOCARTA_AGR_PATHWAY | BIOCARTA_ERK_PATHWAY |
| BIOCARTA_GPCR_PATHWAY | BIOCARTA_PKC_PATHWAY |
| BIOCARTA_ALK_PATHWAY | BIOCARTA_CDMAC_PATHWAY |
| BIOCARTA_SHH_PATHWAY | BIOCARTA_DREAM_PATHWAY |
| BIOCARTA_BARR_MAPK_PATHWAY | BIOCARTA_INTEGRIN_PATHWAY |
| BIOCARTA_NO1_PATHWAY | BIOCARTA_CK1_PATHWAY |
| BIOCARTA_CALCINEURIN_PATHWAY | BIOCARTA_NFAT_PATHWAY |
| BIOCARTA_NOS1_PATHWAY | BIOCARTA_AGPCR_PATHWAY |
|  | |

**ESM. 6:** 14 pathways associated with FGFR1 overexpression in GEO20685

| **List of Pathways which show significance with Up-Regulation of FGFR1 in GEO20685.** | |
| --- | --- |
| BIOCARTA_WNT_PATHWAY | BIOCARTA_GHRELIN_PATHWAY |
| BIOCARTA_TEL_PATHWAY | BIOCARTA_TGFB_PATHWAY |
| BIOCARTA_PS1_PATHWAY | BIOCARTA_RECK_PATHWAY |
| BIOCARTA_ALK_PATHWAY | BIOCARTA_SHH_PATHWAY |
| BIOCARTA_PRION_PATHWAY | BIOCARTA_IGF1MTOR_PATHWAY |
| BIOCARTA_HES_PATHWAY | BIOCARTA_MTA3_PATHWAY |
| BIOCARTA_MYOSIN_PATHWAY | BIOCARTA_P38MAPK_PATHWAY |
|  | |

**ESM.7**: Common pathways in both datasets which are significant with over expression of FGFR1.

| **TCGA DATASET** | | | | | | | | | |
| --- | --- | --- | --- | --- | --- | --- | --- | --- | --- |
| **GS** | | **SIZE** | **ES** | **NES** | **NOM p-val** | **FDR q-val** | **FWER p-val** | **RANK AT MAX** | **LEADING EDGE** |
| 1 | BIOCARTA_PRION_PATHWAY | 12 | 0.72 | 1.91 | 0.002 | 0.076 | 0.089 | 2468 | tags=50%, list=13%, signal=57% |
| 2 | BIOCARTA_ALK_PATHWAY | 36 | 0.62 | 1.86 | 0 | 0.06 | 0.169 | 5477 | tags=67%, list=28%, signal=92% |
| 3 | BIOCARTA_SHH_PATHWAY | 16 | 0.69 | 1.82 | 0.004 | 0.081 | 0.238 | 1560 | tags=31%, list=8%, signal=34% |
| **GSE20685 DATASET** | | | | | | | | | |
| **GS** | | **SIZE** | **ES** | **NES** | **NOM p-val** | **FDR q-val** | **FWER p-val** | **RANK AT MAX** | **LEADING EDGE** |
| 1 | BIOCARTA_PRION_PATHWAY | 11 | 0.73 | 1.68 | 0.016 | 0.338 | 0.655 | 3580 | tags=64%, list=18%, signal=78% |
| 2 | BIOCARTA_ALK_PATHWAY | 37 | 0.53 | 1.67 | 0.008 | 0.286 | 0.658 | 4284 | tags=41%, list=22%, signal=52% |
| 3 | BIOCARTA_SHH_PATHWAY | 16 | 0.56 | 1.49 | 0.053 | 0.569 | 0.975 | 3289 | tags=31%, list=17%, signal=37% |

**ESM. 8:** Positions of Gene Set Members of SHH pathways on the Rank Ordered List figure 1 which shows that these gene sets correspond to the same biological processes while FGFR1 is overexpress.

|  | A) TCGA dataset | | | | | B) GSE20685 | | | | |
| --- | --- | --- | --- | --- | --- | --- | --- | --- | --- | --- |
|  | **PROBE** | **RANK IN GENE LIST** | **RANK METRIC SCORE** | **RUNNING ES** | **CORE ENRICHMENT** | **PROBE** | **RANK IN GENE LIST** | **RANK METRIC SCORE** | **RUNNING ES** | **CORE ENRICHMENT** |
| 1 | GLI2 | 555 | 9.808 | 0.1506 | Yes | GLI1 | 758 | 2.005 | 0.1531 | Yes |
| 2 | PTCH1 | 586 | 9.697 | 0.3259 | Yes | PTCH1 | 984 | 1.82 | 0.3156 | Yes |
| 3 | GLI1 | 1240 | 7.723 | 0.4335 | Yes | GLI3 | 1189 | 1.661 | 0.4639 | Yes |
| 4 | GLI3 | 1259 | 7.69 | 0.5729 | Yes | GLI2 | 2149 | 1.216 | 0.5315 | Yes |
| 5 | PRKAR2B | 1560 | 6.992 | 0.6851 | Yes | PRKAR2B | 3289 | 0.857 | 0.5556 | Yes |

**ESM. 9:** Clinical Analysis and Correlation of FGFR1 and GLI genes with pathological parameters in dataset

|  | **TCGA** | | | |
| --- | --- | --- | --- | --- |
| **Genes** | **FGFR1** | **SHH Pathway Genes** | | |
|  |  | **GLI1** | **GLI2** | **GLI3** |
| **Clinical Features** | **Z-score / p-value** | | | |
| **T-Stage** | - | 36.18/0.0001^b^ | 15.189/0.002^b^ | 11.185/0.011^b^ |
| **Lymph Node** | 14.327/0.002^b^ | 8.087/0.044^b^ | - | - |
| **S-Stage** | -2.387/0.017 ^a^ | 25.552/0.0001 ^b^ | - | - |
| **Metastasis** | - | -2.283/0.022 ^a^ | - | - |
| **Age** | - | -4.894/0.0001 ^a^ | -5.86/0.0001 ^a^ | - |
| **ER +/-** | -5.186/0.0001 ^a^ | -4.951/0.0001 ^a^ | - | -15.738/0.0001 ^a^ |
| **PR +/-** | -2.941/0.003 ^a^ | -5.617/0.0001 ^a^ | -1.943/0.052 | -13.943/0.0001 ^a^ |
| **HER2 +/-** | -2.163/0.031 ^a^ | - | - | -2.032/0.042 ^a^ |
| ^a^ Mann-Whitney Test  ^b^Kruskal-Wallis Test | | | | |

| **Primer name** | **Forward primer** | **Reverse primer** | **Product length (bps)** |
| --- | --- | --- | --- |
| **SHH** | CTTCCTCACTTTCCTGGACCG | GGTGGCCGAGTCGTTGT | 136 |
| **GLI1** | CACATCCACAGCCTCTCTTT | CCTGGGTTCTGAAGGAAGATAAT | 110 |
| **FGFR1** | GATGGAGGTGCTTCACTTAAGA | CATCTTGTAGACGATGACCGAC | 232 |
| **Ki-67** | GCCTTGGTCTCTTGGGAATAC | GGAGATTAGGAGCCAGTTTGAG | 123 |
| **ER** | CCACCAACCAGTGCACCATT | GGTCTTTTCGTATCCCACCTTTC | 108 |
| **PR** | ATTACCAGTGTTCCCGTCTTC | CCTGTACTTCCTCCAGCATAA | 111 |
| **HER-2** | TTGAGTCCATGCCCAATCC | GTGTTCCATCCTCTGCTGTC | 150 |
| **β-actin** | ATGATATCGCCGCGCTCA | CGCTCGGTGAGGATCTTCA | 150 |

**ESM.10:** Information of Primers used for qRT-PCR

**ESM. 11:** Antibodies used in the study

| **Antibody Name** | **Primary Antibody** | **Secondary Antibody** |
| --- | --- | --- |
| **SHH** | (H-160): sc-9024, Santa Cruz Biotechnology, Inc. | Rabbit |
| **GLI-1** | (H-300): sc-20687, Santa Cruz Biotechnology, Inc. | Rabbit |
| **FGFR1** | EPR806Y, Abcam | Rabbit |
| **β-actin** | (C4): sc-47778, Santa Cruz Biotechnology, Inc | Mouse |

**ESM. 12:** Clinical Analysis and Correlation of FGFR1 and GLI genes with pathological parameters in GSE20685 dataset

| **DATASET** | **GSE20685** | | | | | |
| --- | --- | --- | --- | --- | --- | --- |
| **Genes** | **FGFR1** | **SHH Pathway Genes** | | | | |
|  |  | **GLI1** | **GLI2** | **GLI3** | **PTCH1** | **PRKAR2B** |
| **Clinical Features** | **Z-score / p-value** |  |  |  |  |  |
| **Tumor Stage** | - | 7.935/0.047^b^ | - | - | - | 8.864/.031^b^ |
| **Neoplasm Disease lymph Note Stage** | - | - | - | - | - | - |
| **Neoplasm Disease Stage** | - | - | - | - | - | -2.397/.017^a^ |
| **Metastasis Stage** | - | - | - | - | - | - |
| **Age Group** | - | - | - | - | -2.794/.005^a^ | - |
| ^a^ Mann-Whitney Test  ^b^Kruskal-Wallis Test | | | | | | |
